# Simultaneous high expression of PLD1 and Sp1 predicts a poor prognosis for pancreatic ductal adenocarcinoma patients

**DOI:** 10.18632/oncotarget.12447

**Published:** 2016-10-04

**Authors:** Jiong Hu, Hai Hu, Jun-jie Hang, Hai-yan Yang, Zhi-yong Wang, Lei Wang, Dong-hui Chen, Li-wei Wang

**Affiliations:** ^1^ Department of Medical Oncology, Shanghai General Hospital of Nanjing Medical University, Shanghai 201620, China; ^2^ Department of Medical Oncology and Pancreatic Cancer Center, Shanghai General Hospital, Shanghai Jiao Tong University School of Medicine, Shanghai 201620, China; ^3^ Shanghai Key Laboratory of Pancreatic Diseases, Shanghai 201620, China

**Keywords:** PDAC, PLD1, Sp1, prognosis, immunohistochemistry

## Abstract

Pancreatic ductal adenocarcinoma (PDAC) is a lethal disease with few therapeutic options. Recently, insight into cancer biology suggested abnormal lipid metabolism to be a risk factor for human malignancies. As a key enzyme implicated in lipid metabolism, PLD1 was elevated in various human cancer associating with malignant phenotypes. However, little was known about its expression and function in PDAC. We showed that PLD1 was elevated in both the cell lines and clinical samples of PDAC, and it positively correlated with vascular invasion (*p* = 0.041) and responsible for a poor prognosis (*p* = 0.009). Meanwhile, we also found Sp1 to be elevated in the disease, correlating with vascular invasion (*p* = 0.007). Moreover, the correlation assay suggested that PLD1 positively correlated with Sp1 in the clinical sample (*r* = 0.390; *p* < 0.001) and the cell lines. Finally, we showed that co-high expression of both the factors confers the poorest prognosis for the patients, and that their simultaneous high expression might be an independent prognostic factor (*p* = 0.001; HR = 3.427; 95% CI 1.629−7.211).

## INTRODUCTION

Pancreatic ductal adenocarcinoma (PDAC) is a lethal disease with the 5-year survival rate of less than 5% and the median survival of about 6 months, rendering it the fourth most lethal cancer in the United States [1], a frustrating situation had not been changed for decades. Major causes for the disappointing situation include high propensity of early distant metastasis and chemoresistence [2, 3]. However, the molecular and cellular mechanisms underlying these processes remain illusive.

Recently, mounting evidence had suggested abnormal lipid metabolism of the cancer cells to be pro-tumoral in various human cancers [4]. PLD1 was a key enzyme implicated in lipid metabolism by catalyzing the hydrolysis of phosphatidylcholine. As stated, PLD1was elevated in most human malignancies associating with malignant phenotypes. For instance, the prior studies indicated that PLD1 was upregulated in cancers of the intestinal [5] and breast [6]. Functionality analysis showed that elevated PLD1 had a positive correlation with angiogenesis, invasion and distant metastasis as well as chemoresistence of human cancer [7, 8]. Despite of the advancement, little was known about its expression and biological significance in PDAC.

Sp1 is a basal transcription factor belonging to the krüppel-like factor family, and it expressed in nearly all cells of an individual and responsible for proliferation, division, and differentiation [9, 10]. As to cancer, Sp1 was found to be elevated in most tumors responsible for unfavorable phenotypes via transcription activation [11, 12]. For stance, it had been reported that elevated Sp1 contributes to overexpression of multiple oncogenic genes in human cancers [13], including PDAC [14]. For example, Bae IH and colleagues showed that Bcl-w promotes gastric cancer cell invasion by inducing matrix metalloproteinase-2 expression via phosphoinositides 3-kinase, Akt, and Sp1 [15]. Consistently, there was also report that Celecoxib inhibits VEGF expression and reduces angiogenesis and metastasis of pancreatic cancer via the suppression of Sp1 [14]. Since PLD1 was also reported as an oncogenic gene in various human cancers, we boldly postulated that it positive correlated with Sp1, and they could promote PDAC progression synergistically.

In the present study, the expression and the biological significance of PLD1 were investigated. We showed that PLD1 was elevated in PDAC, and it positively correlated with vascular invasion and poor survival. Meanwhile, we also showed that Sp1 was elevated in PDAC, and it significantly correlated with vascular invasion. Moreover, we also showed that PLD1 positively correlated with Sp1 in PDAC, and their simultaneous high expression was an independent prognostic factor for the patients.

## RESULTS

### The baseline characteristics of the PDAC patients

The baseline clinicopathlogical characteristics of the PDAC patients enrolled in this study are summarized in Table 1. Of the 77 patients, 51 were male and 26 were female. The median age of the patients was 62 (ranged from 20 to 78). 73 patients were stage I and/or II, while the rest were diagnosed as metastatic disease. 40 patients had cancers of the head and neck of pancreas, while 37 had cancers in the body and tail of pancreas. Notably, 49 patients exhibited nerve invasion and 10 patients showed vascular invasion.

**Table 1:** The baseline characteristics of PDAC patients

Characteristics	Categories	Number (%)
Gender	Male	51 (66.2)
	Female	26 (33.8)
Age median (range)		62 (20–78)
T stage	T1	4 (5.2)
	T2	16 (20.8)
	T3	57 (74.0)
N stage	N0	35 (45.5)
	N1	42 (54.5)
M stage	M0	73 (94.8)
	M1	4 (5.2)
TMN	IA	4 (5.2)
	IB	10 (13.0)
	IIA	20 (26.0)
	IIB	39 (50.6)
	IV	4 (5.2)
Primary tumor location	Head and Neck	40 (51.9)
	Body and Tail	37 (48.1)
Nuclear grade	I	9 (11.7)
	II	42 (54.5)
	III	26 (33.8)
Nerve invasion	Yes	49 (63.6)
	No	28 (36.4)
Vascular invasion	Yes	10 (13.0)
	No	67 (87.0)

### PLD1 was elevated in pancreatic cancer

**Figure 1:**
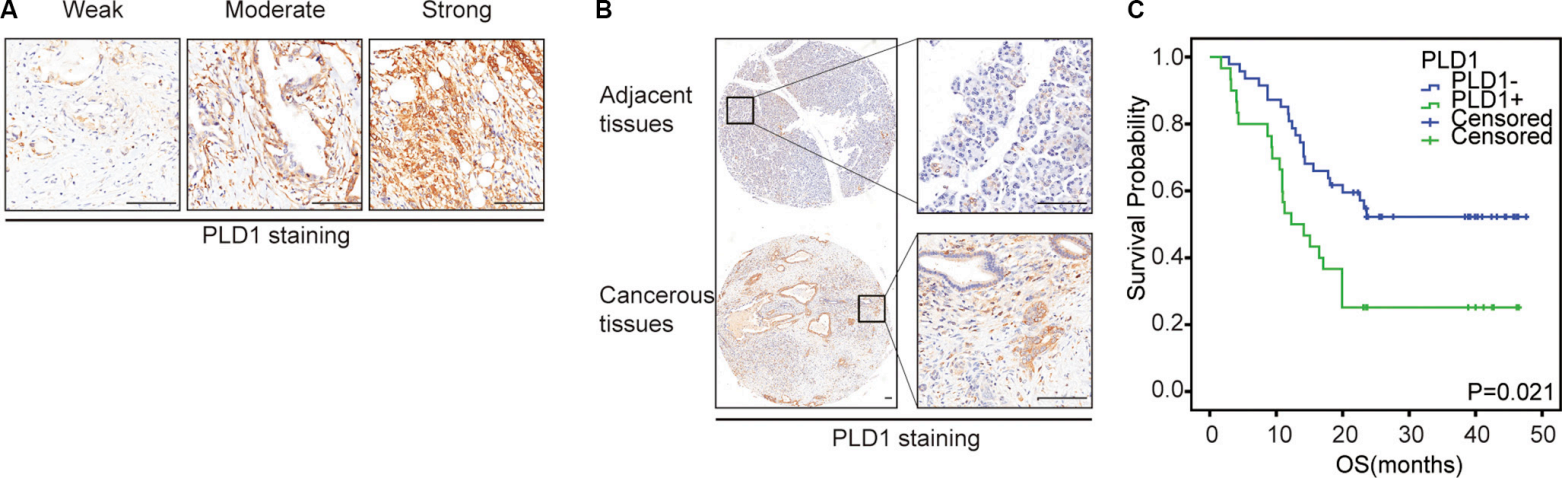
Representative immunohistochemical staining of Sp1 (A-C) and PLD1 (D-E) in PDAC (A, D): Weak positive staining; (B, E): Moderate positive staining; (C, F): Strong positive staining. Bar: 100 um.

To examine the biological significance of PLD1 in PDAC, IHC were used to determine its expression in the tumors. We found that their staining in the patients ranged from weak to strong (Figure 1A). Additionally, we also found a significant difference of PLD1 expression between the cancerous tissues and the paired normal tissues (Figure 1B). Subsequently, we differentiated PLD1 positive patients from their negative counterparts and found that half of the patients were PLD1 positive (Figure 3). Moreover, the correlation assay showed that PLD1 was significantly higher in patients with vascular invasion (*p* = 0.041) compared to those without vascular invasion (Table 2). However, no obvious significance could be observed with other parameters of the patients. Finally, we showed in the survival analysis that PLD1 indicated a poor prognosis (*p* = 0.021, Figure 1C).

**Table 2:** Correlation between Sp1, PLD1 and clinicopathologic features of PDAC patients

Factor	Number	Sp1			PLD1		
		Negative	Positive	*P*	Negative	Positive	*P*
Gender							
Male	51	27 (52.9)	24 (47.1)	0.573	31 (60.8)	20 (39.2)	0.949
Female	26	12 (46.2)	14 (53.8)		16 (61.5)	10 (38.5)	
Age							
> 60	51	27 (52.9)	24 (47.1)	0.573	30 (58.8)	21 (41.2)	0.577
< 60	26	12 (46.2)	14 (53.8)		17 (65.4)	9 (34.6)	
T stage							
T3	57	29 (50.9)	28 (49.1)	0.946	37 (64.9)	20 (35.1)	0.239
< T2	20	10 (50.0)	10 (50.0)		10 (50.0)	10 (50.0)	
N stage							
N0	35	20 (57.1)	15 (42.9)	0.298	23 (65.7)	12 (34.3)	0.443
N1	42	19 (45.2)	23 (54.8)		24 (57.1)	18 (42.9)	
M stage							
M0	73	37 (50.7)	36(49.3)	0.979	44 (60.3)	29 (39.7)	0.951
M1	4	2 (50.0)	2(50.0)		3 (75.0)	1 (25.0)	
TMN stage							
> II	63	31 (49.2)	32 (50.8)	0.591	39 (61.9)	24 (38.1)	0.741
I	14	8 (57.1)	6 (42.9)		8 (57.1)	6 (42.9)	
Primary tumor location							
Head and Neck	40	17 (42.5)	23 (57.5)	0.137	23 (42.5)	17 (57.5)	0.508
Body and Tail	37	22 (59.5)	15 (40.5)		24 (64.9)	13 (35.1)	
Nerve invasion							
Yes	49	24 (49.0)	25 (51.0)	0.698	30 (61.2)	19 (38.8)	0.965
No	28	15 (53.6)	13 (46.4)		17 (60.7)	11 (39.3)	
Vascular invasion							
Yes	10	1 (10.0)	9 (90.0)	0.007	3 (30.0)	7 (70.0)	0.041
No	67	38 (56.7)	29 (43.3)		44 (65.7)	23 (34.3)	
Nuclear grade							
III	26	11 (42.3)	15 (57.7)	0.296	13 (50.0)	13 (50.0)	0.156
< II	51	28 (54.9)	23 (45.1)		34 (66.7)	17 (33.3)	

### Sp1 was elevated in pancreatic cancer

**Figure 2:**
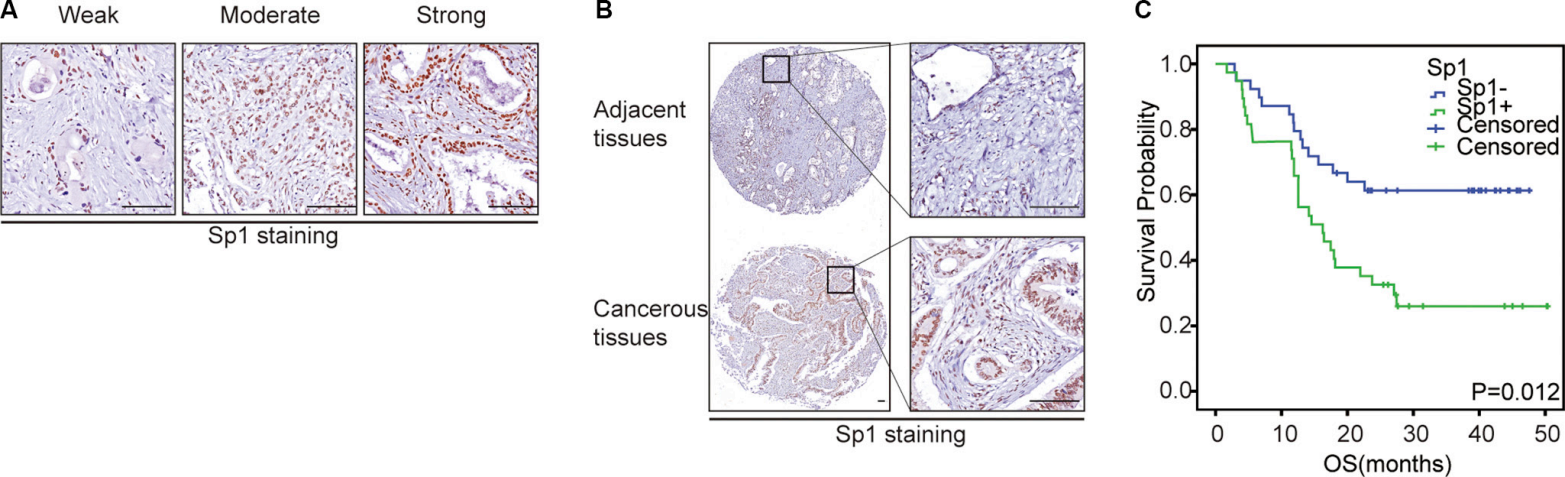
The expression pattern of Sp1 and PLD1 in PDAC (**A**): The expression pattern of Sp1 and PLD1 based on intensity and percentage of the stained cells in PDAC. (**B**) Representative images of PDAC tissues with positive/negative Sp1 and PLD1 expression. Bar: 100 μm.

**Figure 3:**
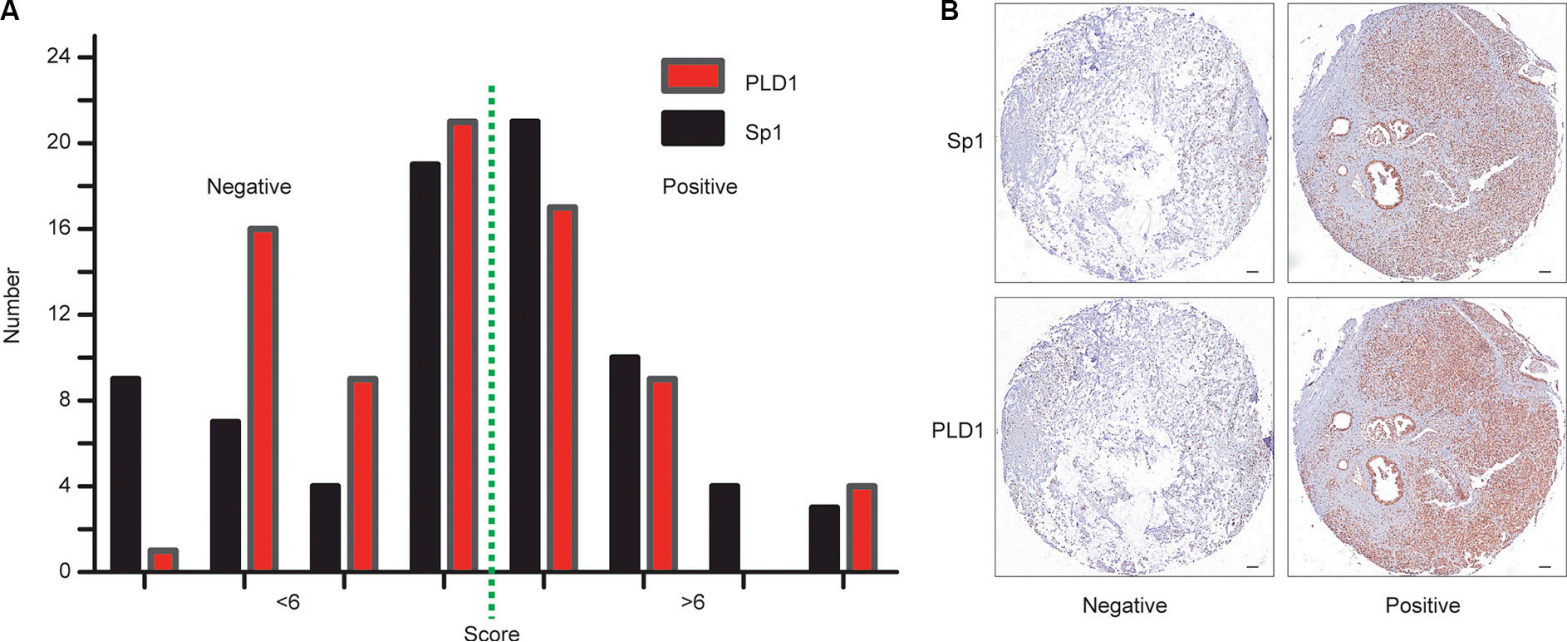
Correlation between Sp1 and PLD1 expression in PDAC samples and cell lines (**A**) The picture depicted the co-distribution of Sp1 and PLD1 in PDAC samples. Spearman's rank correlation coefficient demonstrated a significant correlation between Sp1 and PLD1 (*r* = 0.390; *P* < 0.001). (**B**) showed the PLD1 expression upon Sp1 deletion.

Meanwhile, we also investigated Sp1 expression in PDAC using IHC. As shown in Figure 2A, we found that the staining of Sp1 ranged from negative to strong, with half the patients positive were positive PLD1 staining (Figure 3). Additionally, we also showed a significant difference of Sp1 staining between the cancerous tissues and the paired none cancerous tissues (Figure 2B). Furthermore, the correlation assay showed that Sp1 positively correlated with vascular invasion (*p* = 0.007, Table 2); while no obvious significance could be observed with other parameters of the patients. Finally, the survival analysis showed that Sp1 also confers a poor prognosis for the patients (*p* = 0.012, Figure 2C).

### Correlation between PLD1 and Sp1 in PDAC tissues

**Figure 4:**
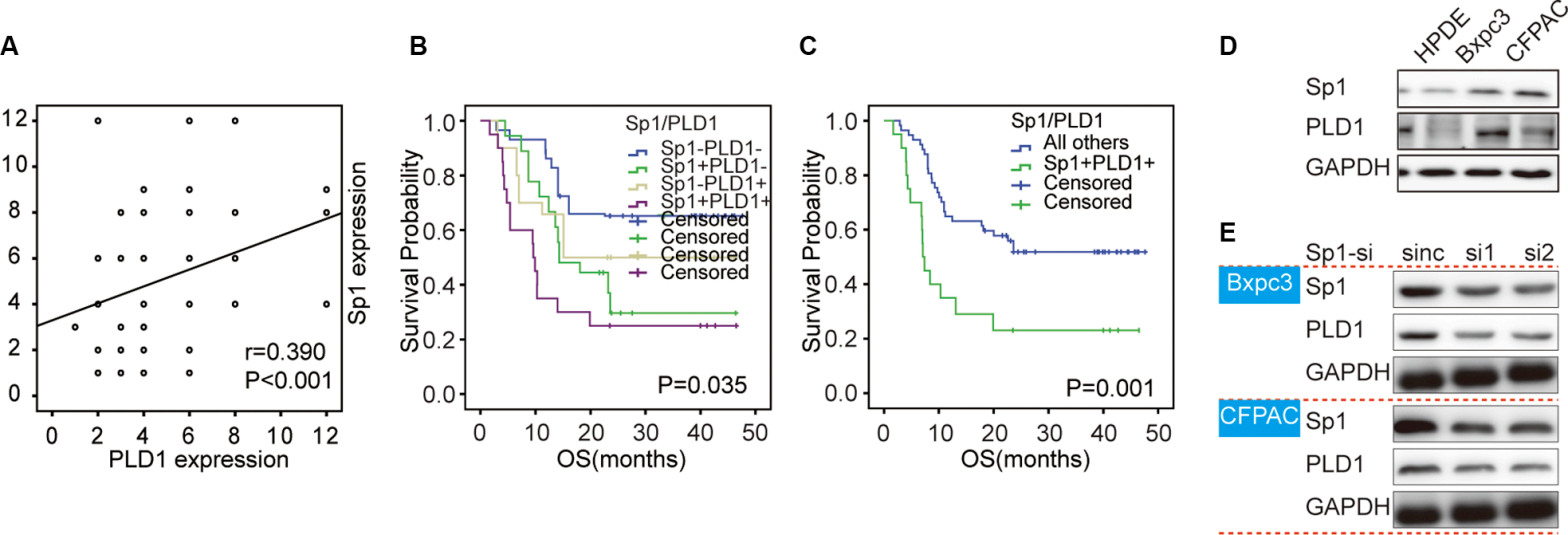
Overall survival curves based on Sp1 and PLD1 expression in PDAC The overall survival curves were based on Sp1 (**A**), PLD1 (**B**), and the combination of Sp1 and PLD1 **(C** and **D**) (as polytomous variables and binary categorical variables, respectively). All others: Sp1^+^PLD1^−^, Sp1^−^PLD1^+^, and Sp1^−^PLD1^−^.

Since both Sp1 and PLD1 contribute to the aggressive of various human cancers, we postulated that they were correlated in the disease. To attain this, serial sections of the same PDAC tissues were scored for stained Sp1 and PLD1 respectively. The final scores of the patients were used to conduct correlation assay, and the data showed that Sp1 is positively correlated with PLD1 (*r* = 0.390; *p* < 0.001, Figure 4A). Moreover, we showed in the Kaplan–Meier assay that combined expression of the two factors confers the poorest prognosis among all the patients (*p* = 0.001, Figure 4B, 4C). Finally, we showed in the multivariate analysis that the combined high expression of Sp1 and PLD1 was an independent prognostic factor for the patients (*p* = 0.001; HR = 3.427; 95% CI 1.629–7.211, Table 3).

**Table 3:** Univariate and multivariate survival analysis of clinicopathologic variables in PDAC patients

Factor	OS median (range)	Univariate analysis			Multivariate analysis		
		HR	95% CI	*P*	HR	95% CI	*P*
Gender							
Male	19.0 (1.0−47.0)	1.054	0.555−2.004	0.871			
Female	20.0 (3.0−46.0)	1					
Age							
> 60	22.0 (1.0−47.0)	0.677	0.365−1.254	0.215			
< 60	15.5(2.0−46.0)	1					
T stage							
T3	17.8 (1.7−47.6)	1.778	0.822−3.845	0.144			
< T2	23.0(3.0−46.0)	1					
N stage							
N0	22.3 (2.9−47.6)	1.279	0.694−2.357	0.431			
N1	17. (1.7−45.8)	1					
M stage							
M0	18.4 (1.7−47.6)	0.658	0.175−3.005	0.658			
M1	23.2 (12.4−40.3)	1					
TMN stage							
> II	15.6 (1.7−47.6)	3.052	1.087−8.567	0.034	3.223	1.044−9.951	0.042
I	31.1 (12.6−46.5)	1			1		
Primary tumor location							
Head and Neck	16.4 (1.7−47.6)	1.204	0.657−2.209	0.548			
Body and Tail	23.1 (2.9−46.4)	1					
Nerve invasion							
Yes	13.6 (1.7−46.5)	2.171	1.089−4.328	0.028	1.573	0.738−3.356	0.241
No	23.7 (3.1−47.6)	1			1		
Vascular invasion							
Yes	7.9 (3.1−46.5)	2.958	1.352−6.473	0.007	2.560	0.977−6.705	0.056
No	22.3(1.7−47.6)	1			1		
Nuclear grade							
III	12.1(1.7−44.6)	2.374	1.290−4.368	0.005	2.725	1.391−5.341	0.003
< II	23.2(4.0−47.6)	1			1		
Sp1							
Positive	12.5(1.7−46.5)	2.599	1.377−4.903	0.003			
Negative	23.7(2.9−47.6)	1					
PLD1							
Positive	9.4(1.7−46.5)	2.212	1.203−4.069	0.011			
Negative	23.2(2.9−47.6)	1					
Sp1/PLD1							
Sp1+/PLD1+	7.3(1.7−46.5)	2.768	1.463−5.238	0.002	3.427	1.629−7.211	0.001
All others	23.1(2.9−47.6)	1			1		

Additionally, we also found in the multivariate analysis that TNM stage (*p* = 0.042; HR = 3.223; 95% CI 1.044–9.951), and nuclear grade (*p* = 0.003; HR = 2.725; 95% CI 1.391–5.341) were independent prognostic factors for PDAC patients (Table 3).

### Correlation between PLD1 and Sp1 in PDAC cell lines

To further confirm the positive correlation between in Sp1 and PLD1 in PDAC, we investigated their expression and correlation in pancreatic duct epithelial (HPDE) cells and pancreatic cancer cell. As shown in Figure 4D, we found that both Sp1 and PLD1 were elevated in cancer cell lines compared to HPDE. Subsequently, we knockout Sp1 expression of the cancer cells and found that PLD1 decreased concomitantly (Figure 4E). Taken together, our data showed that Sp1 positive correlated with PLD1 in PDAC.

## DISCUSSION

There is growing interest in understanding the role that abnormal metabolism in the initiation and progression of cancer. In this study, we had focused on the role of PLD1, a key enzyme implicated in lipid metabolism, in PDAC. Our results show that PLD1 was elevated in the disease and it correlated with vascular invasion. More importantly, our data further showed that Sp1 is elevated in PDAC, and it positively correlated with PLD1 with that their simultaneous overexpression predicts a poor prognosis for patients.

PLD1 functions to catalyze the hydrolysis of phosphatidylcholine PC so as to generate phosphatidic acid (PA), substrates associating with various signaling cascades, such as Wnt, mTOR, and NF-kB [7, 16, 17]. As stated, PLD1 were reported to be overexpression in various human tumors and contribute to the malignant phenotypes of human cancers, such as angiogenesis, invasion and metastasis, and chemoresistence [8, 18, 19]. In the present study, we reported for the first time that PLD1 is overexpression in PDAC. Our data also revealed that PLD1 is closely correlated with vascular invasion, suggesting that PLD1 might also involve in local invasion and distant metastasis of PDAC. Taken together, the data highlights a critical role of PLD1 in PDAC, and that targeted inhibition of PLD1 might be a novel direction for the management of PDAC.

Sp1 is a ubiquitously expressed nuclear transcription factor with three zinc fingers in the C-terminal domains that binds to the GC/GT box of target genes [20]. Under normal conditions, Sp1 was expressed in all cells of an individual responsible for cell propagation, differentiation and division. As stated, it is also overexpression in most human cancers and functions to stimulate angiogenesis, invasion and metastasis as well as chemoresistence by upregulating the relevant genes expression [21–23]. For instance, Xie and colleagues [24] found that Sp1 could transcriptionally activate VEGF expression, a molecule associates with angiogenesis and distant metastasis in PDAC. Moreover, some researchers proposed early in 1993 that Sp1 was responsible for chemoresistence; since they showed that Sp1 could transcriptionally activate MDR expression [25]. Consistently, our data also showed that Sp1 was positively correlated with vascular invasion in PDAC, suggesting that it might also involved in distant metastasis of PDAC.

Previously, researchers had established the positive correlation for Sp1 and other genes, they showed that there was a zinc finger in the C-terminal of Sp1, and it is the very structure that binds to the GC/GT boxes of the target genes leading to their expression [26]. In the present study, we showed that Sp1 positively correlated with PLD1, and that their simultaneous high expression confers the poorest prognosis for the patients. These data showed that the two factors might collaborate with each other so as to promote PDAC progression. Hence, further studies were needed to clarify whether transcription activation also apply to the positive correlations between Sp1 and PLD1 in PDAC. In addition, some of the prior studies indicated that PLD1 functions upstream of Wnt/beta-catenin [27] and JAK/STAT3 [28] signaling, which subsequently phosphorylate the downstream molecules. As phosphorylated Sp1 was the active form of Sp1; we, therefore, proposed another hypothesis to link them together, which suggested that PLD1-triggered signaling could subsequently activate Sp1 by phosphorylation in PDAC.

In conclusion, our findings have revealed strong expression of PLD1 and Sp1 in PDAC. In addition, our data showed that Sp1 positively correlated with PLD1 in the lethal disease. Statistical analysis demonstrated that both Sp1 and PLD1 correlated closely with vascular invasion and their simultaneous overexpression confers a poor prognosis for patients. Since PLD1 serves as an oncogenic protein in PDAC, further studies are needed to determine its full function and regulation so as to reveal novel therapeutic targets for the lethal disease.

## MATERIALS AND METHODS

### Patients

77 patients with histopathologic diagnosis of primary PDAC (ICD, Tenth Revision, codes C25) were included in our study. Pancreatic cancerous tissues and adjacent paired normal tissues were collected from the department of pathology at Shanghai Jiaotong University Affiliated Shanghai General Hospital, Shanghai, China from 2012 to 2014. The last follow-up visit was on February 28th, 2016. The patients' clinicopathological characteristics include age, gender, TNM stage, primary tumor location, nerve invasion, vascular invasion, and nuclear grade (Table 1). Each patient provided written informed consent and the study approved Ethics Committees of Shanghai General Hospital.

### Tissue microarray construction

The microarray was made as described [29]. Briefly, H&E-stained sections were made from primary tumor blocks to define tumor regions. Representative tumor regions are defined as areas with at least 75% cancer cells without necrosis. Tissue cylinders (1.5 mm in diameter) were then punched from the regions of the block using a tissue microarrayer (Gentury, IL, USA) and placed into recipient paraffin blocks. Sections of the TMA blocks were transferred to glass slides.

### Immunohistochemistry (IHC)

The standard IHC protocol has been described previously [21]. In brief, the tissue microarrays were dewaxed and dehydrated in xylene and alcohol bath solutions, respectively. Endogenous peroxidase activity was then blocked using 0.3% hydrogen peroxide for 10 mins, before antigen retrieval was undertaken by setting the slides in 0.01 M citrate buffer (pH 6.0) at 98°C for 5 min using a microwave oven. The slides were cooled to room temperature and blocked by incubating them with normal goat serum at room temperature for 1 h, followed by incubation at 4°C overnight with the primary antibodies (Cell Signaling Technology, Beverly, MA, USA). Finally, the sections were incubated with HRP-labeled secondary antibody and visualized using diaminobenzidine.

### Evaluation of IHC

Evaluation of the staining was performed by two independent pathologists blind to research in at five areas at 400× magnification. The staining was scored according to the intensity and percentage of the stained cells. Staining intensity was assigned as 0 (no staining), 1 (weak staining), 2 (moderate staining), and 3 (strong staining). The percentages were classified into: 1 (≤ 25%), 2 (25%–50%), 3 (50%–75%), and 4 (75%–100%). The final scores were calculated as the staining intensity × the percentage of positive cells. For statistical analyses, a score < 6 was regarded as negative expression, and > 6 as positive expression. The dilution of the primary antibodies: Sp1 (1:100); PLD1 (1:100).

### Cell lines and cell culture

Human pancreatic duct epithelial (HPDE) cells and pancreatic cancer cell lines were purchased from Shanghai Institute for Life Science, Chinese Academy of Sciences. All the cells were cultured in RPMI 1640 supplemented with 10% fetal bovine serum (FBS, Gibco, Carlsbad, CA, USA) at 37°C in a humidified atmosphere of 95% air and 5% CO2, and grown in a humidified atmosphere of air/CO2 (95%: 5%). Cells with gene deletion/overexpression were cultured in the same condition with 1.5-μg/mL puromycin (Sigma-Aldrich, St. Louis, MO, USA).

### Establishment of PDAC cell lines with short hairpin RNA (shRNA)

The GV-248 lentiviral RNAi expression system (Genechem, Shanghai, China) was used to prepare the lentivirus expressing human Sp1 shRNA. Sp1-expression plasmids were constructed by cloning the cDNA encoding Sp1 into the system. The targeting sequences were, Sp1-Si1: 5′-GCAGTACCAATGGCAGCAATG-3′; Sp1-Si2: 5′-GCAGACCTTTACAACTCAA-3′. The scramble sequences was 5′-TTCTCCGAACGTGTCACGT-3′. The polyclonal cells with puromycin resistance were selected for subsequent experiments. The protocol of transfection had been described previously [30].

### Western blot analysis

Cells were washed three times with cold PBS and lysed on ice in RIPA buffer with protease inhibitors PMSF (Beyotime Biotechnology, China). Protein concentrations were determined by BCA method (Beyotime Biotechnology, China). A total of 20 μg protein was separated by 10% SDS-PAGE and electro-blotted onto NC membranes using semi-dry blotting apparatus. After blocking in 3% bovine serum albumin (BSA), the membranes were incubated with the primary antibodies overnight at 4°C. The membranes were washed and incubated with the secondary antibodies for 1h at room temperature on a shaker. The protein bands were visualized using a commercially available enhanced chemiluminesence kit (Thermo Scientific, Hudson, NH, USA). GAPDH were used as control. The primary antibodies used in the study include: Sp1 (1:1000), PLD1 (1: 1000) (CST, Beverly, MA, USA); and GAPDH (Santa Cruz Biotechnology, CA, USA).

### Statistical analysis

Statistical analyses were performed using SPSS software (version 21.0; SPSS Inc, Chicago, IL, USA). The relationships between the clinicopathlogical factors and Sp1/PLD1 expression were investigated using Pearson χ^2^ test. The Spearman's rank test was used to evaluate their correlation. Kaplan–Meier analysis was used to demonstrate differences in overall survival (OS). The correlation between the prognostic factors and OS was investigated with the Cox regression model. Results were considered statistically significant when *p* < 0.05.

## Abbreviation

Pld1: phospholipase D1
Sp1: Specificity protein-1.
